# Vitamin D Deficiency in Dengue Hemorrhagic Fever and Dengue Shock Syndrome among Sri Lankan Children: A Case-Control Study

**DOI:** 10.1155/2021/4173303

**Published:** 2021-10-14

**Authors:** Sharmila Dissanayake, Sureshi Tennekoon, Sharmila Gaffoor, Guwani Liyanage

**Affiliations:** ^1^Paediatric Professorial Unit, Colombo South Teaching Hospital, Kalubowila, Sri Lanka; ^2^Department of Paediatrics, Faculty of Medical Sciences, University of Sri Jayewardenepura, Nugegoda, Sri Lanka

## Abstract

**Introduction:**

Dengue fever is a vector-borne disease associated with a significant public health impact. The clinical picture ranges from undifferentiated fever to more severe forms such as dengue hemorrhagic fever (DHF) and dengue shock syndrome (DSS). Compared to healthy controls, we explored the likelihood of having vitamin D deficiency (VDD) among children with severe dengue infection.

**Methods:**

This case-control study compared hospitalized children (2 months to 12 years) with DHF and DSS with radiologically confirmed plasma leak with age-matched healthy controls. The association of 25-hydroxy vitamin D [25(OH)D] level, age, sex, and socioeconomic status with DHF/DSS was assessed using univariate and multivariate logistic regression.

**Results:**

Forty children with DHF/DSS were compared with 52 healthy controls. Mean (SD) age was 8.8 (2.9) years and 7.9 (3.7) years among cases and controls, respectively. Most (*n* = 28, 70%) had DHF. In multivariate logistic regression, the likelihood of having VDD [25(OH)D < 20 ng/mL] was 3.6 times higher in cases compared to controls (Odds Ratio (OR): 3.65, 95% Confidence Interval (CI): 1.461, 9.102, *p*=0.006). When serum 25(OH)D was used as a continuous independent variable, the strength of the association between DHF/DSS and serum 25(OH)D was weak but statistically significant; the likelihood of having DHF/DSS is 0.94 times less with 1 ng/mL increase in serum 25(OH)D (OR: 0.940, 95% CI: 0.887, 0.995, *p*=0.03).

**Conclusion:**

The present study suggests that the likelihood of having VDD among children with DHF/DSS is higher than that in their healthy counterparts. Thus, further studies are critical in confirming whether vitamin D repletion is beneficial in preventing severe forms of dengue in the quest to reduce the morbidity and mortality associated with dengue infection.

## 1. Introduction

Dengue infection has been endemic in Sri Lanka since the mid-1960s [1]. However, a dramatic increase in incidence was observed during the last decade, attributed to urbanization and population growth [2]. Therefore, seasonal and periodic epidemics are prevalent in urban and suburban areas. The highest number of reported dengue cases in 2019 was from the Colombo district (20,718 out of 105,049 cases in the country) [3]. The case fatality rate in the same year was approximately 0.09%. According to recent statistics, the incidence is the highest among people aged 20–29 years [4]. In children and young adults below 20 years of age, the incidence is higher among the 10–19-year-olds than that in the younger age group (64%). However, younger children are at a higher risk of severe dengue than older children and adults [5].

A vaccine to prevent dengue is licensed and available in some countries, recommended for children aged nine years and above [6]. Several other vaccine candidates are also under development for children above four years of age [6, 7]. In the absence of a specific antiviral therapy, the management is primarily supportive. Vitamin D is thought to help reduce the disease severity. It probably lowers the number of infected cells, especially monocytic cells, with subsequent reduction of proinflammatory cytokines such as tumor necrosis factor-alpha (TNF-*α*), interleukin-6 (IL-6), and interleukin-1beta (IL-1*β*) [8]. Yet, there is conflicting evidence on the association of serum vitamin D levels with dengue infection [9–11]. Furthermore, only very few studies have assessed the association of vitamin D with more severe disease, particularly among infants and young children, despite severe disease being commoner among younger than older children. Therefore, we explored the likelihood of having vitamin D deficiency among children (2 months to 12 years) with DHF/DSS compared to healthy controls in an urban setting.

## 2. Materials and Methods

### 2.1. Study Design

A case-control study of hospitalized children with dengue hemorrhagic fever was carried out in a single center in Sri Lanka from July 2019 to January 2020. The Colombo South Teaching Hospital is situated in the Colombo district and provides a wide range of secondary and tertiary services to an urban catchment population where dengue infection is prevalent. In our study, the potential participants included all the children aged two months to 12 years with physician-diagnosed dengue hemorrhagic fever, admitted for in-ward care over seven months. The sample size calculation used the formula (*n* = 2 (*Zα*/2 + *Zβ*)2 (*σ*2/*d*2) with *α* = 0.05 and *β* = 0.2 (80% power). According to a previous study, the population standard deviation for 25(OH)D was taken as 6 ng/mL among 10–18–year-old Sri Lankan children [12]. We expected to see a difference between means, that is, 70% the size of the standard deviation (*d* = 4.2 ng/mL). Thus, the minimum sample size calculated was 32 for each group.

Children with serologically confirmed dengue infection were screened, and all with DHF/DSS were recruited consecutively over the study period. Based on caregiver reports and clinic records, children with chronic diseases were excluded (children with kidney, liver, and bone disease and children on long-term medications that affect bone metabolism). The control subjects were enrolled by convenience sampling from the same hospital within at least one week as the case for comparison of vitamin D status. Thus, controls were considered coming from the same catchment population as the cases and were loosely matched for age (<5 years, 5–9 years, and ≥10 years). Children with chronic illness, bone deformities, or a documented history of DHF/DSS were not included in the control sample.

### 2.2. Ethics Statement

The study was conducted based on the rules of the Declaration of Helsinki and approved by the Ethics Committee of Postgraduate Institute of Medicine, Colombo (ERC/PGIM/2019/131). Informed written consent was taken from the caregivers before data collection. In addition, oral assent was taken for children above seven years of age.

### 2.3. Definitions

Case definitions of dengue infection were based on published guidelines [13, 14]. Dengue hemorrhagic fever was defined as fever of 2–7 days, the presence of bleeding manifestations or positive tourniquet test, low platelet count (≤100,000 cells/mm^3^), and objective evidence of plasma leakage (ultrasound scan evidence ascites and/or pleural effusion). For children who had definite evidence of plasma leakage, the presence of hemorrhagic manifestations was not essential to diagnose DHF. DSS was defined as DHF with circulatory failure (cold extremities, prolonged capillary refill time >2 secs, and narrow pulse pressure (<20 mm Hg)). The presence of serological evidence of dengue infection was considered when nonstructural protein-1 antigen assay by immunofluorescence (<5 days) or IgM antibody by enzyme-linked immunosorbent assay (≥5 days) was positive [15]. Vitamin D status was classified as a serum 25(OH)D level of >30 ng/mL was sufficient, 20–30 ng/mL was insufficient, and <20 ng/mL was deficient [16].

### 2.4. Procedure

As per the unit protocol, children with suspected or confirmed dengue infection were monitored with clinical, laboratory parameters, and serial in-ward ultrasound scans for disease progression. An ultrasound scan abdomen confirmed the earliest indication of entry into the leakage phase. If the ward scan findings were doubtful, a confirmatory scan was performed by the on-call radiologist. A blood sample (3 ml) for vitamin D assessment was collected when the leaking was confirmed. Children with circulatory instability and DSS at baseline were not included. The investigators were not involved in the clinical management of the study subjects. Serum was stored at −20 to −80°C until analysis. Serum total 25-(OH) D was analyzed with the LIAISON 25 (OH) Vitamin D TOTAL assay kit (DiaSorin Inc. USA) in batches, and results were not available for patient management. Total circulating 25(OH)D [25(OH)D2 and 25(OH)D3] is considered as the acceptable biomarker for serum vitamin D status [17]. According to the manufacturer, it has an analytical range of 4–150 ng/ml with and an acceptable intra-assay and interassay variability. Cross reactivity is reported as 104% to 25-OHD2 and 100% to 25-OHD3.

Sociodemographic and clinical details were collected. Furthermore, additional details were taken from the patient records (white cell count (WBC), platelet count, aspartate aminotransferase (AST), alanine aminotransferase levels (ALT) activity, and crystalloid, colloid, or blood transfusions). The lowest WBC and platelet count out of the serial measurements of each participant were recorded. Similarly, details of sociodemography, present and past health, and a blood sample (3 ml) were collected from the control subjects.

### 2.5. Data Analysis

We used the Statistical Package for the Social Sciences (SPSS), version 22 software package for data analysis. The normality of data was checked. Descriptive statistics are expressed as frequency, percentages, mean, or median, as necessary. Comparisons between cases and controls were conducted with the chi-squared test and Mann–Whitney U test. The association of VDD, age, sex, and socioeconomic proxy variable (family income) with DHF/DSS was assessed with univariate analysis (unconditional logistic regression). For logistic regression analysis, serum 25 (OH)D level was taken as a continuous variable and categorical variable (<20 ng/mL and ≥20 ng/mL; <30 ng/mL and ≥30 ng/mL). These thresholds were chosen using established definitions of vitamin D status [16]. Age was included in the regression analysis as age was loosely matched between cases to controls [18]. All variables with a *p* value less than 0.2 were included in the multivariate analysis.

## 3. Results

**Figure 1:**
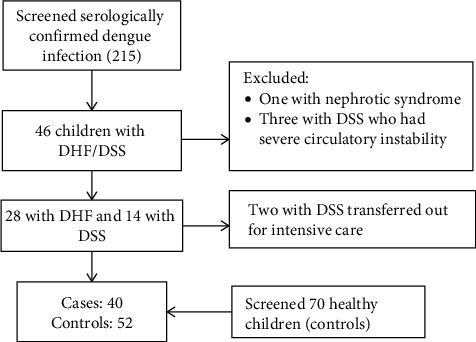
Algorithm for subject enrolment during the study period.

Forty-six children with severe dengue infection were identified. Four were excluded (Figure 1). The response rate was 95% for cases; data collection could not be completed in two patients as they were transferred out (Figure 1). Seventy health children were invited as controls, and the response rate was 74.3%. Reasons for dropouts were refusal to consent (*n* = 14) and hemolyzed samples (*n* = 4). Baseline characteristics of cases and controls are given in Table 1. Forty children with DHF/DSS were compared with 52 healthy controls. Mean (SD) age was 8.8 (2.9) years and 7.9 (3.7) years among cases and controls, respectively. Most (70%) had DHF. All children with DHF/DSS required intravenous crystalloid transfusions, and sixteen children required colloid solutions. In addition, three children were given blood transfusions. No difference in age, sex, and family income was found between the cases and controls. However, the frequency of vitamin D deficiency was significantly higher among cases than in controls (*p*=0.01). Mean (SD) serum 25(OH)D among cases and controls was 19.9 ng/mL (7.8) and 23.2 ng/mL (7.9), respectively.

A comparison between vitamin D-deficient and -nondeficient groups was carried out (Table 2). The mean 25(OH)D levels showed a significant difference between the groups among cases and controls.

Univariate logistic regression taking serum 25(OH)D as a continuous independent variable did not show a significant difference between cases and controls (OR:0.946, 95% CI:0.895, 1.001, *p*=0.06). When serum 25 (OH)D level was taken as a categorical variable (<20 ng/mL and ≥20 ng/mL), cases had higher odds of having deficiency (OR:2.97, 95% CI:1.261, 7.00, *p*=0.01). However, with 25(OH)D <30 ng/mL and ≥30 ng/mL, a difference between controls and cases was not observed (OR:2.10, 95% CI: 0.673, 6.551, *p*=0.20). Only predictors with low *p* values ≤of 0.2 were considered for multivariate regression. Three models were derived, taking age and vitamin D as independent variables (Table 3). In the model considering 25 (OH)D as a continuous variable, adjusted for age, the association between serum 25(OH)D and DHF/DSS was significant, but the strength of the association was low; the likelihood of having DHF/DSS is 0.94 times less with 1 ng/mL increase in serum 25(OH)D. When categorical variables were used in the model, the likelihood of having VDD was more than three times higher among the DHF/DSS. However, no association was found when serum 25 (OH)D was categorized as <30 ng/mL vs. ≥30 ng/mL.

## 4. Discussion

In this case-control study, the likelihood of having VDD was higher among children with DHF/DSS compared to healthy controls. The possible association of VDD with severe dengue infection could be explained in several ways. First, it is known that severe dengue is linked to high cytokine concentrations released by T cells, monocytes/macrophages, and endothelial cells in response to high viral loads [19]. Second, vitamin D supports macrophage differentiation, thus restricting viral replication [20]. Also, it has been shown that cytokine production is significantly lower in dengue-infected macrophages with vitamin D treatment than that without [20].

The available evidence on the link between vitamin D status and dengue infection is scanty and inconsistent [9–11]. Fatima et al. reported low vitamin D status among adults with dengue fever (DF) compared to unmatched healthy controls [9]. The sample included a case-to-control ratio of 1 : 1 (15 subjects in each group), and vitamin D level was analyzed using a spectrophotometric method. It is important to keep in mind that problems of accuracy of laboratory methods could lead to systematic errors and, thus, may cause variability of research findings. Alagarasu et al., in a recent study in India, reported contrasting results [10]. The study compared vitamin D levels in adults with DF (*n* = 83) and DHF (*n* = 29) with healthy individuals. The adults with DF/DHF had higher serum 25(OH)D levels than healthy controls, which was statistically significant [10]. Among the adults with DHF in that study, only six had objective evidence of plasma leak (ascites and/or as pleural effusion). In comparison, in the present study, all the children had radiological evidence of plasma leak.

Villamor et al. studied the association of initial vitamin D status at the febrile phase and its relationship with the progression of the disease to DHF/DSS among children and adults [11]. The study concluded that low serum 25 (OH)D levels during the febrile phase predicted decreased odds of progression to DHF/DSS. Overall, these inconsistencies among studies may be explained by differences in case definitions, dengue virus serotypes, age of the participants, and disease severity.

The study findings should be interpreted with the following limitations. Although case-control studies can be used to establish a relationship between exposures and outcomes, they cannot establish causation. In the present study, children with DHF/DSS were compared with healthy children without documented evidence of dengue infection. However, the quality of the findings could have been improved by including another comparison group of children with mild disease (DF). We conducted the study in a single center and found a weak relationship between DHF/DSS and vitamin D. Furthermore, future studies could recruit multiple controls to each case to increase the study's statistical power. Also, a multicenter study over a longer period would have given clearer results which are more convincing, as the patient sample of multicenter studies is supposed to be representative. Although cases and controls were considered as coming from the same catchment population, matching for confounders such as environment variables (viz., area of residence) could have probably increased the validity of the results more.

## 5. Conclusions

The present study suggests that the likelihood of having VDD among children with DHF/DSS is higher than that in their healthy counterparts. Further studies are critical in confirming whether vitamin D repletion is beneficial in preventing severe forms of dengue in the quest to reduce the morbidity and mortality associated with dengue infection.

## Figures and Tables

**Table 1:** Characteristics of the study sample.

Characteristics	Cases, *n* (%) (*n* = 40)	Controls, *n* (%) (*n* = 52)	*p* value
Age, years, mean (SD)	8.8 (2.9)	7.9 (3.7)	0.20
Sex (male)	22 (55)	25 (48)	0.51
Family income, LKR/month, median (IQR)	50 (100–150)	57.5 (20–100)	0.05
Serum 25(OH)D <20 ng/mL	26 (65)	20 (38.4)	0.01
Serum 25(OH)D <30 ng/mL	35 (87.5)	40 (76.9)	0.19

Expressed as *n* (%) unless otherwise stated. LKR: Sri Lankan Rupees. For comparisons, ANOVA, the chi-squared test, and the Mann–Whitney U test were used as appropriate.

**Table 2:** Comparison of vitamin D-deficient (<20 ng/mL) and -nondeficient (≥20 ng/mL) groups among cases and controls.

	Cases		*p* value	Controls		*p* value
	25(OH)D < 20 ng/mL	25(OH)D ≥ 20 ng/mL		25(OH)D < 20 ng/mL	25(OH)D ≥ 20 ng/mL	
Number	26	14		20	32	
Age, yrs (mean, SD)	8.2 (2.9)	10.1 (2.3)	0.04	7.1 (3.7)	8.4 (3.7)	0.226
Sex (male), *n* (%)	14 (53.8)	8 (57.1)	0.55	9	16	0.726
25(OH)D (ng/mL), mean (SD)	15.4 (3.4)	28.4 (6.61)	<0.001	16.5	27.5	<0.001
WBC (10^9^/L)	3.8 (1.6, 6.2)	3.2 (1.1, 6.5)	0.34	—	—	—
Platelets (10^9^/L)	33 (10, 86)	37.5 (10, 90)	0.94	—	—	—
ALT (U/L)	42 (21, 475)	48.5 (14, 249)	0.88	—	—	—
AST (U/L)	113 (49, 409)	105 (42, 459)	0.52	—	—	—
DSS, *n* (%)	6 (23.7)	6 (42.9)	0.19	—	—	—
Blood transfused, *n* (%)	2 (7.7)	1 (7.1)	0.54	—	—	—

AST- aspartate aminotransferase, ALT- alanine aminotransferase, DSS- dengue shock syndrome, WBC- white cell count. Values are expressed as median (range) unless otherwise indicated. For comparisons, ANOVA, the chi-squared test, and the Mann–Whitney U test were used as appropriate.

**Table 3:** Models derived with multivariate logistic regression analysis of associated factors of DHF/DSS.

Model	Adjusted OR	95.0% CI for adjusted OR		*p* value
		Lower	Upper	
^*∗*^25(OH)D (continuous variable)	0.94	0.887	0.995	0.03
^*∗∗*^25(OH)D <20 ng/mL vs. ≥20 ng/mL	3.65	1.461	9.102	0.006
25(OH)D <30 ng/mL vs. ≥30 ng/mL	2.33	0.730	7.431	0.15

^*∗*^Log-likelihood ratio = 119.4, Nagelkerke *R*^2^ = 0.09, chi squared (*X*^2^) = 6.6, *p*=0.04. ^*∗∗*^Log-likelihood ratio = 116.0, Nagelkerke *R*^2^ = 0.14, *X*^2^ = 9.9, *p*=0.007.

## Data Availability

Data are available from the corresponding author on a reasonable request.
